# miR-92b-3p protects retinal tissues against DNA damage and apoptosis by targeting BTG2 in experimental myopia

**DOI:** 10.1186/s12967-024-05288-3

**Published:** 2024-05-28

**Authors:** Jinpeng Liu, Bo Bao, Tuling Li, Zhaohui Yang, Yongle Du, Ruixue Zhang, Jizhao Xin, Jiawen Hao, Guimin Wang, Hongsheng Bi, Dadong Guo

**Affiliations:** 1 Shandong University of Traditional Chinese Medicine, Jinan, 250014 China; 2 Affiliated Eye Hospital, Shandong University of Traditional Chinese Medicine,No. 48#, Yingxiongshan Road, Jinan, Shandong 250002 China; 3 Shandong Provincial Key Laboratory of Integrated Traditional Chinese and Western Medicine for Prevention and Therapy of Ocular Diseases, Experimental Center, Shandong Academy of Eye Disease Prevention and Therapy, Medical College of Optometry and Ophthalmology, Shandong University of Traditional Chinese Medicine, No. 48#, Yingxiongshan Road, Jinan, Shandong 250002 China

**Keywords:** BTG2, Lens-induced myopia, Retina, miR-92b-3p, Apoptosis

## Abstract

**Background:**

Myopia is one of the eye diseases that can damage the vision of young people. This study aimed to explore the protective role of miR-92b-3p against DNA damage and apoptosis in retinal tissues of negative lens-induced myopic (LIM) guinea pigs by targeting BTG2.

**Methods:**

Biometric measurements of ocular parameters, flash electroretinogram (FERG), and retinal thickness (RT) were performed after miR-92b-3p intravitreal injection in LIM guinea pigs. The apoptotic rate was detected by Annexin V-FITC/PI double staining, and the change in mitochondrial membrane potential was measured by JC-1 staining. Retinal apoptosis and expression of p53, BTG2, and CDK2 were explored by TdT-mediated dUTP-biotin nick labeling (TUNEL) and immunofluorescence staining assays, respectively. BTG2 and its upstream and downstream molecules at gene and protein levels in retinal tissues were measured by real-time quantitative PCR (qPCR) and Western blotting.

**Results:**

Compared with normal controls (NC), the ocular axial length of LIM guinea pig significantly increased, whereas refraction decreased. Meanwhile, dMax-a and -b wave amplitudes of ERG declined, retinal thickness was decreased, the number of apoptotic cells and apoptotic rate in LIM eyes was exaggerated, and the mitochondrial membrane potential significantly decreased. In addition, results of qPCR and Western blot assays showed that the expression levels of p53, BTG2, CDK2, and BAX in LIM guinea pigs were higher than the levels of the NC group, whereas the BCL-2 expression level was decreased. By contrast, the miR-92b-3p intravitreal injection in LIM guinea pigs could significantly inhibit axial elongation, alleviate DNA damage and apoptosis, and thus protect guinea pigs against myopia.

**Conclusion:**

In conclusion, p53 and BTG2 were activated in the retinal tissue of myopic guinea pigs, and the activated BTG2 could elevate the expression of CDK2 and BAX, and attenuate the expression of BCL-2, which in turn promote apoptosis and eventually lead to retinal thinning and impaired visual function in myopic guinea pigs. The miR-92b-3p intravitreal injection can attenuate the elongation of ocular length and retinal thickness, and inhibit the CDK2, BAX, and p53 expression by targeting BTG2, thereby ameliorating DNA damage and apoptosis in LIM guinea pigs and protecting ocular tissues.

**Graphical Abstract:**

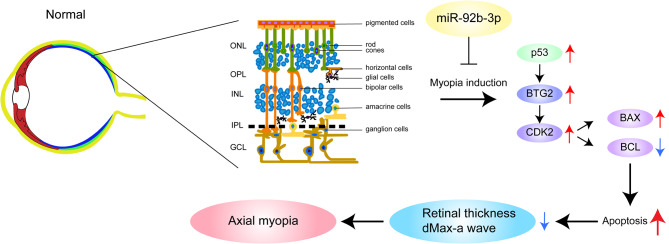

## Background

According to epidemiological surveys, myopia has become a common eye disease worldwide, with a noteworthy prevalence of 80–90% in East Asian adolescents [1]. The complications of high myopia can lead to severe visual impairment, which can result in retinal photoreceptor dysfunction and photoreceptor death, thus causing visual impairment and even blindness in myopic patients [2]. Most cases of myopia are associated with the rapid growth of the eye axis, and the mechanism of the growth of the eye axis is mainly related to environmental and genetic factors [3]. The retina is an important tissue in the eye, in which cone cells and rod cells play a key role in light presentation, but the mechanism by which myopia affects retinal functional feedback is still unclear [4]. In the early stages of myopia, the thickness of the retinal nerve fiber layer is reduced, especially around the optic papilla [5]. But the exact cause of apoptosis due to apoptosis is still unclear. In contrast, PrimPol in highly myopic individuals may lead to mutations that also cause the generation of apoptosis. In addition, DNA instability and DNA repair defects are associated with the onset and development of retinal neuronal degeneration [6]. The accumulation of DNA breaks caused by external factors also usually occurs inside nerve cells, and blue light has been shown to induce DNA breaks in retinal cells both in vitro and in vivo [7, 8]. 

BTG2 acts as an anti-proliferative protein and its protein product is implicated in many cellular processes, including cell division, DNA repair, transcriptional regulation, and messenger RNA stability. In addition to influencing differentiation during development and adulthood, BTG proteins play a vital role in maintaining homeostasis under conditions of cellular stress [9]. Moreover, the BTG2 gene is overexpressed in the G0/G1 phase of the cell cycle of neuroepithelial cells, which suggests a role in the induction of nerve growth and differentiation [10]. BTG2 is required for DNA damage to induce G2/M blockade, resulting in apoptotic effects, and it can regulate the activity of p53 through post-translational modifications, thereby mediating the transition from senescence to apoptosis due to p53 regulation [11]. 

MicroRNAs (miRNAs) are a family of small endogenous RNAs capable of post-transcriptional regulation of gene expression, which can inhibit mRNA translation and reduce mRNA stability by binding to the 3’-untranslated region (3’UTR) of the target mRNA [12]. A previous study has shown that increased expression of miR-92a-3p activates the Wnt/β-catenin pathway, thereby inhibiting the suppression of apoptosis in mitochondria [13]. Meanwhile, BTG2 can also be regulated by many miRNAs, such as miR-21, miR-18, and miR-32, which all result in reduced BTG2 expression, thereby affecting BTG2’s downstream role in regulating cell growth, death, migration, and apoptosis [14, 15]. Thus, BTG2 serves as a significant target involved in regulating a variety of cell biological processes, especially for processes affecting apoptosis.

To investigate the role of BTG2 signaling-related molecules in the retina in the development of myopia, we established the lens-induced myopia (LIM) model in guinea pigs, and then supplemented miR-92b-3p-carrying lentivirus (intravitreal injection) in experimental myopic eyes. After 4- and 6-weeks myopic induction, we measured retinal thickness (RT) and electroretinogram, determined BTG2-related molecular levels in the retinal tissues, and evaluated the effect of miR-92b-3p on DNA damage and apoptosis of retina in LIM guinea pigs. This study aimed to elucidate the effects of DNA damage and apoptosis on the development of myopia, providing new ideas for the treatment of myopia in clinical practice.

## Materials and methods

### Animals

The present study has been approved by the Ethics Committee of Affiliated Hospital of Shandong University of Traditional Chinese Medicine (AWE-2022-055) and strictly followed the Association for Animal Research in Vision and Ophthalmology (ARVO) principles. Animals were placed in a room at 25 °C ± 2 °C with a circadian rhythm of 12 h/12 h (alternating day/night). Before the experiments, all animals were tested to exclude ocular diseases such as cataracts and corneal diseases.

In this study, 120 healthy British tricolor shorthair guinea pigs (male, two weeks old, weighing 100 ~ 120 g, Danyang Chang Yi Experimental Animal Breeding Co., Ltd., China) were randomly divided into a normal control (NC) group, a lens-induced myopia (LIM) group, a LIM + miR-92b-3p-carrying lentivirus (4µL) treatment(LV/4µL) group, a LIM + miR-92b-3p-carrying lentivirus (6µL) treatment(LV/6µL) group, and a LIM + miR-92b-3p-Vector-carrying (6µL) treatment (VECTOR) group. All animals in the NC group were untreated, while those in the LIM, LV/4µL, LV/6µL, and VECTOR groups were covered with a -6.0D lens on the right eye to induce experimental myopia, and the untreated left eye served as self-control. In addition, animals in the LV/4µL and LV/6µL groups were injected with 4µL and 6µL (1E12VG/mL) of miR-92-3p-carrying lentivirus (Genomeditech, Shanghai, China) via the vitreous cavity, respectively; meanwhile, those in the VECTOR group received 6 µL of the vector without miR-92-3p. In addition, we patrolled twice a day (in the morning and the evening) and if the lenses were blurred or detached, they were immediately replaced with clean lenses.

### Dual luciferase-reporter assay and GO enrichment of related genes

**Fig. 1:**
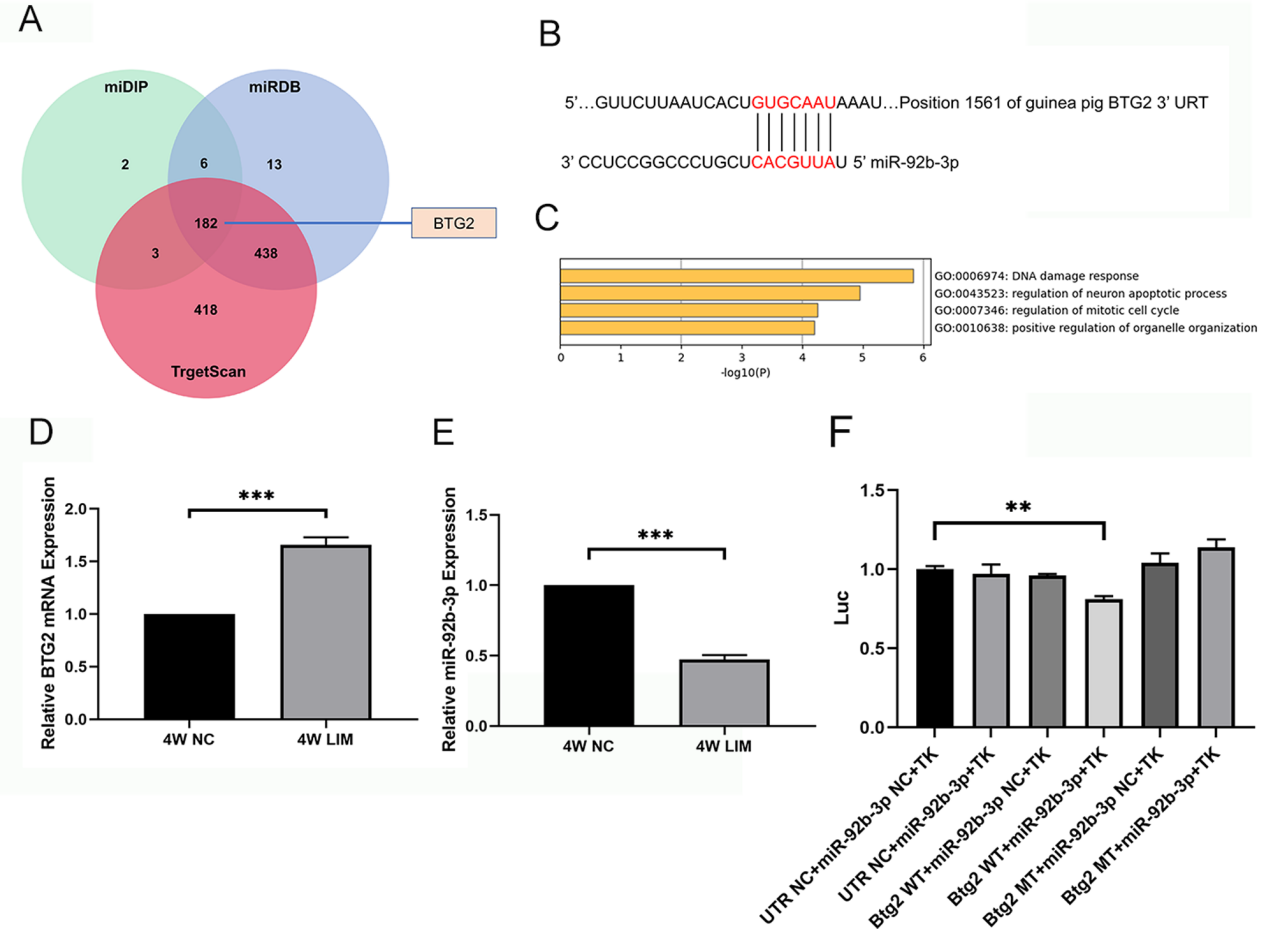
Analysis of miR-92b-3p regulating BTG2. (**A**) Venn diagram of potential targets of miRNA regulation predicted by databases. miDIP (green), miRDB (blue), and Targetscan (red). (**B**) miR-92b-3p regulates BTG2 expression predicted by bioinformatics. (**C**) BTG2-, CDK2-, p53-, BAX-, and BCL2-related signaling pathway enrichment analysis annotated by the Matescape database. (**D**) BTG2 expression levels of the retinal tissues in guinea pigs determined by q-PCR. (**E**) miR-92b-3p expression levels of the retinal tissues in guinea pigs determined by q-PCR. (**F**) Dual luciferase reporter gene assay of miR-92b-3p regulating BTG2 expression. ***P < 0.001 vs. UTRNC + miR-92b-3p NC + TK group

We examined the levels of BTG2 and miR-92b-3p in the retinas of four-week myopic guinea pigs using q-PCR and found that BTG2 in the retinal tissues of the LIM group was elevated whereas miR-92b-3p was decreased compared with the levels of the NC samples (Fig. 1). Based on this finding, we performed a bioinformatics algorithm to predict the binding site of miR-92b-3p to BTG2. Further, a dual-luciferase reporter assay was used to verify whether BTG2 was the target gene regulated by miR-92b-3p. The 3’UTR sequences of the BTG2 gene and its mutant were cloned into the dual luciferase gene vector, and wild-type and mutant recombinant dual luciferase reporter plasmid vectors were constructed. PCR assay and gene sequencing were performed to determine whether the vectors were successfully constructed. Lipofectamine 2000 (Invitrogen) was co-transfected with the 3’-Utrluciferase reporter plasmid and miRNA mimics for luciferase assay. After transfection of HEK-293 cells for 48 h (transfection efficiency ~ 80%), firefly, and renilla luciferase activities were measured using a dual luciferase assay kit (Promega). The renilla luciferase value was divided by the firefly luciferase activity value to normalize the difference in transfection efficiency. The experiment was repeated three times.

To validate the function of the target genes, we entered the target genes used in the study into the Metascape website (http://metascape.org) for GO function enrichment to obtain the relevant function of the target genes [16]. 

### Biometric measurements

Refractive error in each group was measured after 4- and 6-week myopic induction. Before an examination, 10 mg/mL of cyclopentolate hydrochloride eye drops (Alcon, Geneva, Switzerland) were administered into the conjunctival sac of the guinea pigs three times and one drop each time. Then refractive examinations were performed 30 ~ 45 min after the last drop. Each eye was measured at least six times, and the average was regarded as the final experimental result.

Next, we used Quantel Medical measurement (Cournon-d’auvergne, France) to measure the ocular axial length of the guinea pigs. First, one drop of 0.4% oxybuprocaine hydrochloride (Benoxicam, Japan) was used before measurement. Then ocular axial length was measured. The propagation velocity of the anterior chamber was 1557 m/s, the propagation velocity of the lens was 1723 m/s, and that of the vitreous was 1540 m/s.^17^ [17] A total of 10 readings were averaged as the final readings.

### Retinal thickness measurement

**Fig. 2:**
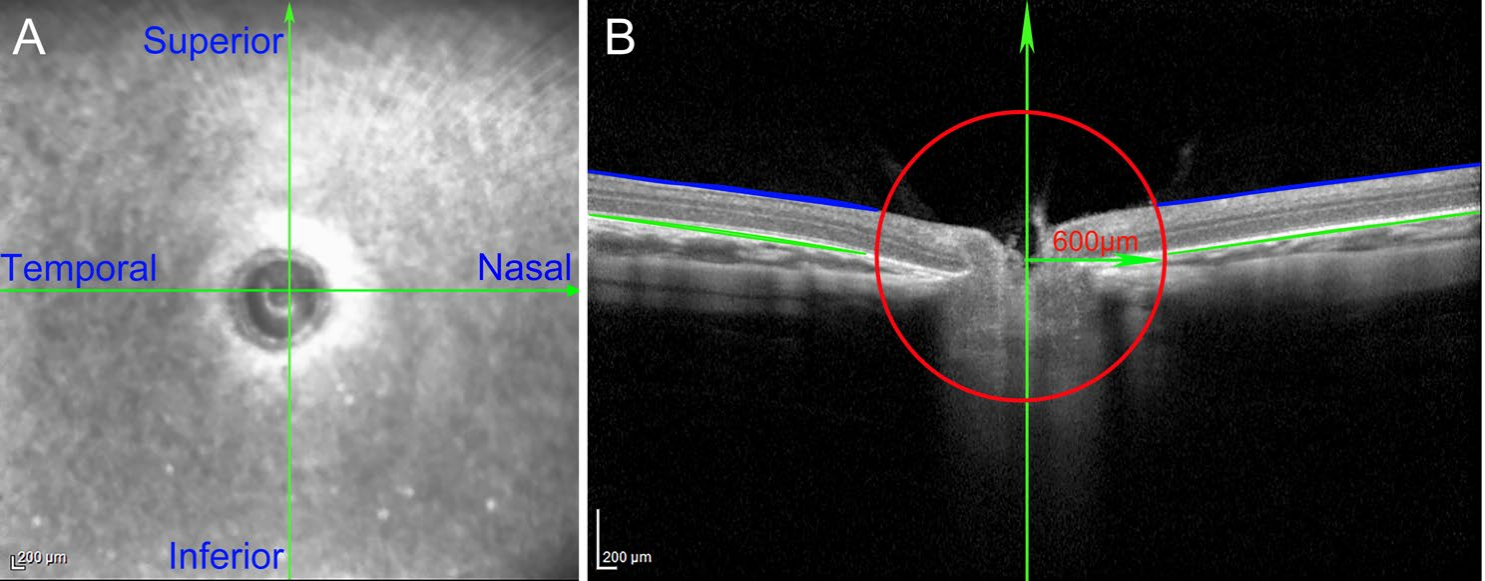
Schematic diagram of the determination of retinal thickness. (**A**) OCT measurement template of the guinea pig fundus. (**B**) Structural images captured by OCT show the structure of the delimited regions; red lines represent the inner concentric circle (600 μm, green arrows). The optic nerve disc is defined as the center of the circle, and the region of interest in each quadrant is located at the boundary of the retinal layer (between the green and blue lines)

We used SD-OCT (Spectral Domain Optical Coherence Tomography Imaging; Heidelberg Engineering, Heidelberg, Germany) to photograph the retina of guinea pigs in each group. Images were taken with the optic nerve papilla as the center of the scan, and OCT images were captured in the inferior, nasal, temporal, and upper quadrants. Referred to the previous literature [18], the total retinal thickness in this study was defined as the thickness from the retinal nerve fiber layer (RNFL) to the retinal pigment epithelium (RPE). As shown in Fig. 2, the thickness of the entire retina includes the thickness between the RNFL and RPE layers, while between the green and blue lines highlighted line marks the location of the retina. The highlighted line marks the location of the RNFL (Blue) and RPE (Green) layers. To analyze the retinal thickness, we measured the average retinal thickness in the inferior, nasal, temporal, and superior quadrants of the optic disc, and the average retinal thickness of the optic disc was as the final retinal thickness (600 μm beyond the optic disc, Fig. 2).

### Flash electroretinogram

To evaluate the function of the whole retina, we recorded flash electroretinograms (FERG) after 6-week treatments using a visual electrophysiology system (Optoprobe OPTO-III, Optoprobe Sciences Ltd., UK). After anesthesia, guinea pigs were placed on an animal manipulation platform and flash responses were recorded through a ring corneal electrode (3.00 mm diameter, gold wire) fixed to the corneal surface while ground electrodes penetrated subcutaneously in the right arm. FERG examination consisted of stimulus intensity of 3 log cd-s/m^2^ with a stimulus interval of 30 s and was used to assess dark-adapted mixed cell responses. FERG a-wave amplitude was defined as the distance from the baseline to the a-wave trough, while the b-wave amplitude was measured as the distance between the trough and the peak. The maximum (Max) wave amplitude was recorded.

### Apoptotic assay

We used Annexin V/PI staining to monitor the apoptosis of the retinal cells. Briefly, after a 6-week myopic induction and treatment with 4µL and 6µL of miR-92b-3p, the fresh retinal tissues were isolated, rinsed with PBS, and then cut the tissues into small pieces. Next, the pieces were then transferred into 15-ml tubes (Nest Biotech., Wuxi, China), adding 3 mL of 1640 medium, 1,350 µL of collagenase I, 375 µL of collagenase IV, and 180 µL of hyaluronidase, followed by digestion for 40 min at 37 °C. At the indicated time, the cell suspensions were collected and centrifuged at 600 g for 5 min. Finally, the cell pellets were resuspended in 1×PBS (pH 7.4) and were divided into two volumes. One was for Annexin V/PI binding assay, and the other was for JC-1 staining assay.

For the Annexin V/PI binding assay, floating and adherent cells were collected and rinsed with PBS, followed by centrifugation at 600 g for 5 min to collect the cells. Next, the cells were then resuspended in 400 µ L of Annexin V-FITC binding buffer at a concentration of 1 × 10^5^ cells/mL, followed by the addition of 5 µL of Annexin V-FITC, and the cells were gently stirred and incubated in the dark at 4℃ for 15 min. Subsequently, another 10 µL of PI solution was added to the cell suspension and incubated for 10 min at 4 ℃ in the dark, and then the cell suspensions were immediately analyzed by flow cytometry (NovoCyte, Agilent, USA).

### Measurement of the mitochondrial membrane potential

In this section, JC-1 staining working solution was added in cell suspensions suspended in 1×PBS (pH7.4), blended gently, and incubated at 37 °C for 20 min. Next, the cell suspensions were washed with JC-1 staining buffer, centrifuged at 600 g for 5 min, and then the cell pellet resuspended with 1 ml of JC-1 staining buffer. Finally, the samples were analyzed using a flow cytometer.

### TUNEL assay

Six weeks after myopic induction, three guinea pigs per group were anesthetized intraperitoneally with 4% sodium pentobarbital. The eyes of the guinea pigs were immediately extracted and rinsed in sterile saline to remove the tissue around the eyes. The eyes were fixed in ocular fixative (G1109, Service bio, Wuhan, China) for 24 h, followed by routine dehydration, paraffin embedding, and sectioning. Paraffin sections were then dewaxed, repaired with proteinase K working solution, incubated in an incubator at 37 °C for 22 min, and then washed with PBS. The tissues were covered with a breaking film working solution and incubated at room temperature for 20 min, washed with PBS, and incubated with PBS for 10 min at room temperature, followed by incubation with the TUNEL reaction solution (G1502, Service bio, Wuhan, China) at 37 °C for 2 h. After washing, slides were closed with anti-fluorescence quenching sealer and then observed under a fluorescence microscope (Nikon, Eclipse, Tokyo, Japan). Nuclei in the tissue stained by DAPI are shown in blue, and positive apoptotic nuclei are shown in green.

### Real-time quantitative PCR

To detect the expression level of miR-92-3p, miRNA was extracted from the retinas of guinea pigs with the miRNA Extraction Kit (Sparkjade Science Co. Ltd., Jinan, China) after 4- and 6-week myopic induction. Reverse transcription was performed using the miRNA First Strand Synthesis Kit (Sparkjade Biotechnology Co. Ltd., Jinan, China). The real-time quantitative PCR (qPCR) conditions were: 94 °C, 5 s, 1 cycle; 94 °C for 5 s, 54 °C for 15 s, 72 °C for 10 s, 45 cycles. The primer sequences are shown in Table 1. The results were analyzed by the 2^−ΔΔ^Ct method [19]. 

**Table 1:** Primer sequences for target genes

Gene		Primer sequences
GAPDH	Forward:	5’-CTG ACC TGC CGC CTG GAG AAACC-3’
	Reverse:	5’-ATG CCA GCC CCA GCG TCA AAAGT-3’
U6	Forward:	5’- -CGCTTCACGAATTTGCGTGTCAT-3’
	Reverse:	5’--GCTTCGGCAGCACATATACTAAAAT-3’
P53	Forward:	5’-GCCATCTACAAGAAGTCACAGCACA -3’
	Reverse:	5’-CCAGGCCATCACTATCGGAACA -3’
BTG2	Forward:	5’-GAGGACCCGAGGCTGCGTGAGTG-3’
	Reverse:	5’-GCTGGGGCTGGCTGAGTCC-3’
CDK2	Forward:	5’- CCGCCTGGACACTGAGACTGAAG -3’
	Reverse:	5’- GGACCCGATGAGAATGGCAAAAT -3’
BAX	Forward:	5’-GCATGGGCGGGGACACTTTG-3’
	Reverse:	5’-GCACAGCGCCTTGAGCACCAG-3’
BCL-2	Forward:	5’-CTCCCGCCGCTATCG CCAAGACT-3’
	Reverse:	5’-GACCCCACCGAACTCAAAGAAGG-3’
miR-92b-3p	Forward:	5’-GCGTATTGCACTCGTCCCG-3’
	Reverse:	5’- AGTGCAGGGTCCGAGGTATT-3’

To detect the expression levels of p53, BTG2, CDK2, BCL-2, and BAX, total RNAs were extracted from the retinal tissues of guinea pigs using a tissue/cell RNA extraction kit (Sparkjade Science Co. Ltd., Jinan, China) after 4- and 6-week myopic induction. Reverse transcription was done using Hiscript II Q RT Supermix for QPCR (+ gDNA wiper) (Vazyme Biotech Co., Nanjing, China) to obtain the cDNA of the target genes. The cDNA of the target genes was analyzed in a 96-well plate (NEST Biotechnology, Wuxi, China) using Chamq Universal SYBR QPCR Master Mix (Vazyme Biotech Co. Ltd., Nanjing, China). Real-time quantitative PCR (qPCR) was performed on the cDNA of the above target genes using Chamq Universal SYBR QPCR Master Mix (Vazyme Biotech Co., Nanjing, China) in 96-well plates (NEST Biotechnology, Wuxi, China). The primers synthesized by Sangon Biotech Biotechnology Co.; Ltd. (Shanghai, China) are shown in Table 1. The relative expression levels of p53, BTG2, CDK2, BCL-2, and BAX genes in the retinas of guinea pigs were detected by qPCR, with GAPDH as the internal reference gene. The results were analyzed by the 2^−ΔΔCt^ method.

### Western blotting

After 4- and 6-week myopic induction, the retinal tissues of guinea pigs in each group were isolated, and then RIPA lysis buffer containing PMSF (Sparkjade Biotechnology Co., Jinan, China) was added. After grinding under liquid nitrogen, the supernatant was obtained by centrifugation at 4 °C and 6000 g for 5 min, and then the total protein concentration of each sample was determined by a BCA assay kit (Sparkjade Biotechnology Co., Ltd., Jinan, China). The proteins were then separated by electrophoresis on 15% gel separation gels and transferred into polyvinylidene fluoride (PVDF) membranes, then blocked with 5% fat-free milk or 5% albumin from bovine serum (BSA, Sparkjade, China) at room temperature for 2 h. Next, the blots were reacted with p-p53 (dilution 1:5000, Proteintech, Wuhan, China), BTG2 (dilution 1:1000, Bioss, Beijing, China), CDK2 (dilution 1:8000, Proteintech, Wuhan, China), BCL-2 (dilution 1:5000, Proteintech, Wuhan, China), Caspase 3(dilution 1:1000, Proteintech, Wuhan, China), Caspase 9(dilution 1:1000, Proteintech, Wuhan, China)and BAX (dilution 1:5000, Proteintech, Wuhan, China) at 4 °C overnight, respectively. Then, the PVDF membrane-loaded target protein was incubated with an HRP-conjugated secondary antibody at 4 °C for 1 h. Finally, the images were captured with a FUSION-FX7 imaging system and quantified with FUSION CAPT software (Vilber Lour Mat, France).

### Immunofluorescence assay

In this section, the experimental procedures were referred to as the “TUNEL staining” section. After completing antigen repair, BSA was washed with PBS (5 min × 3), added dropwise, and closed for 30 min. Next, p-p53 (diluted 1:100, Proteintech, Wuhan, China), BTG2 (diluted 1:300, Bioss, Beijing, China), CDK2 (diluted 1:300, BIOSS, Beijing, China), BCL-2 (diluted 1:200, Proteintech, Wuhan, China) primary antibodies were incubated overnight at 4 °C. In addition, slides were rinsed with PBS and then incubated with HRP-labeled secondary antibody (dilution 1:2000, Proteintech, Wuhan, China) for 50 min at room temperature. At the indicated times, slides were washed with PBS and incubated with DAPI dye solution for 10 min in a dark room. The slides were then sealed with an anti-fluorescent bursting agent and observed with a fluorescence microscope (Nikon Eclipse, 55i, Japan).

### Statistical analysis

Data are expressed as mean ± SD. One-way ANOVA was used for comparison among groups, and an independent samples t-test was used for comparison between the two groups. All analyses were performed with GraphPad Prism 8.0 software. *P* < 0.05 is considered a significant difference.

## Results

### Changes in refraction and axial length

**Fig. 3:**
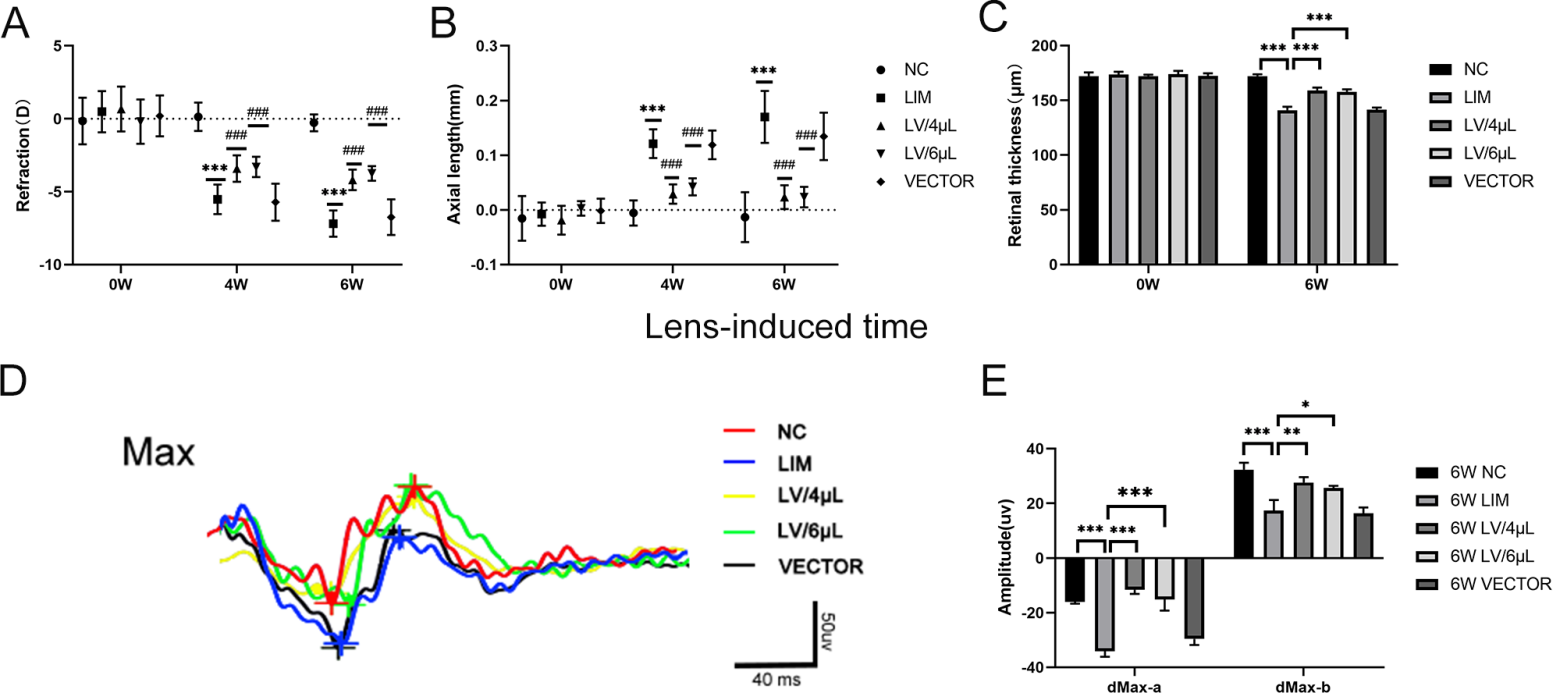
Changes in biological parameters of the eyes in the NC, LIM, LV/4µL, LV/6µL, and VECTOR groups after 4- and 6-week myopic induction. (**A**) Refraction, (**B**) axial length, **(C**) Retinal thickness, (**D**) FERG images, and (**E**) histogram analysis of FERG data. *P < 0.05, **P < 0.01, ***P < 0.001 vs. the NC group; ###*P* < 0.001 vs. the LIM group

After 4-week myopic induction, we assayed the difference in refraction and ocular axial length between the lens-induced myopic eye (right eye) of the LIM group and the ipsilateral eye of the NC group, and we found that the refraction of the LIM eye significantly decreased, whereas the axial length of the LIM eye significantly increased compared with that of the NC group (both *P* < 0.001). However, the refraction of the LV/4µL group was significantly increased compared with that of the LIM group, whereas the ocular axial length significantly decreased (*P* < 0.05), and the refraction and ocular axial length in the LV/6µL group showed the same trend. Nevertheless, there was no significant difference between the LV/4µL treatment group and the LV/6µL treatment group. Meanwhile, results also showed that there was no significant difference in refractive error and ocular axial length between the VECTOR and LIM groups (Fig. 3., *P* > 0.05). Importantly, we also found that after a 6-week myopic induction, it showed the same trend as the 4-week treatment.

### Retinal thickness

We measured the retinal thickness of the animals in each group before enrollment and found that there was no significant difference between either of the two groups (*P* > 0.05). After 6-week myopic induction, we found that compared with the NC group, the retinal thickness of the LIM eye was significantly thinner (Fig. 3, *P* < 0.05). However, after treatment with either 4µL or 6µL of miR-92b-3p-carrying lentivirus, the retinal thickness significantly increased, and there was no significant difference between LV/4µL and LV/6µL treatment groups (Fig. 3). In addition, we also noted that there was no significant difference between the LIM and LIM + EV groups.

### Electroretinography analysis

After a 6-week myopic induction, the dMax-a wave amplitude of FERG significantly reduced in the LIM group compared with that of the NC group **(**Fig. 3, *P* < 0.05), and the b-wave amplitude in both groups showed the same trend as the a-wave. By contrast, after either 4 µL of miR-92b-3p-carrying lentivirus or 6 µL of miR-92b-3p-carrying lentivirus treatment, dMax-a wave amplitude significantly elevated compared with that of the LIM group. Meanwhile, we also found that there was no significant difference in a- and b-wave amplitudes between the VECTOR group and LIM groups (Fig. 3, ^***^P < 0.01).

### Dual luciferase-reporter assay and GO enrichment

We first predicted potential targets for miRNA regulation using the database and validated BTG2 and miR-92b-3p binding sites using Targetscan (Fig. 1).

We used a dual luciferase reporter assay to investigate the relationship between miR-92b-3p and BTG2. Results showed that miR-92b-3p had a significant inhibitory effect on BTG2 wild-type fluorescent plasmid luciferase activity (Fig. 1). However, there was no significant difference in the reporter fluorescence intensity in the mutant vector after mutation at the predicted target site (*P* > 0.05), indicating that BTG2 is a target gene regulated by miR-92b-3p. In addition, the target gene enrichment results also showed an association with DNA damage and apoptosis of neurons (Fig. 1).

### Apoptosis assay

**Fig. 4:**
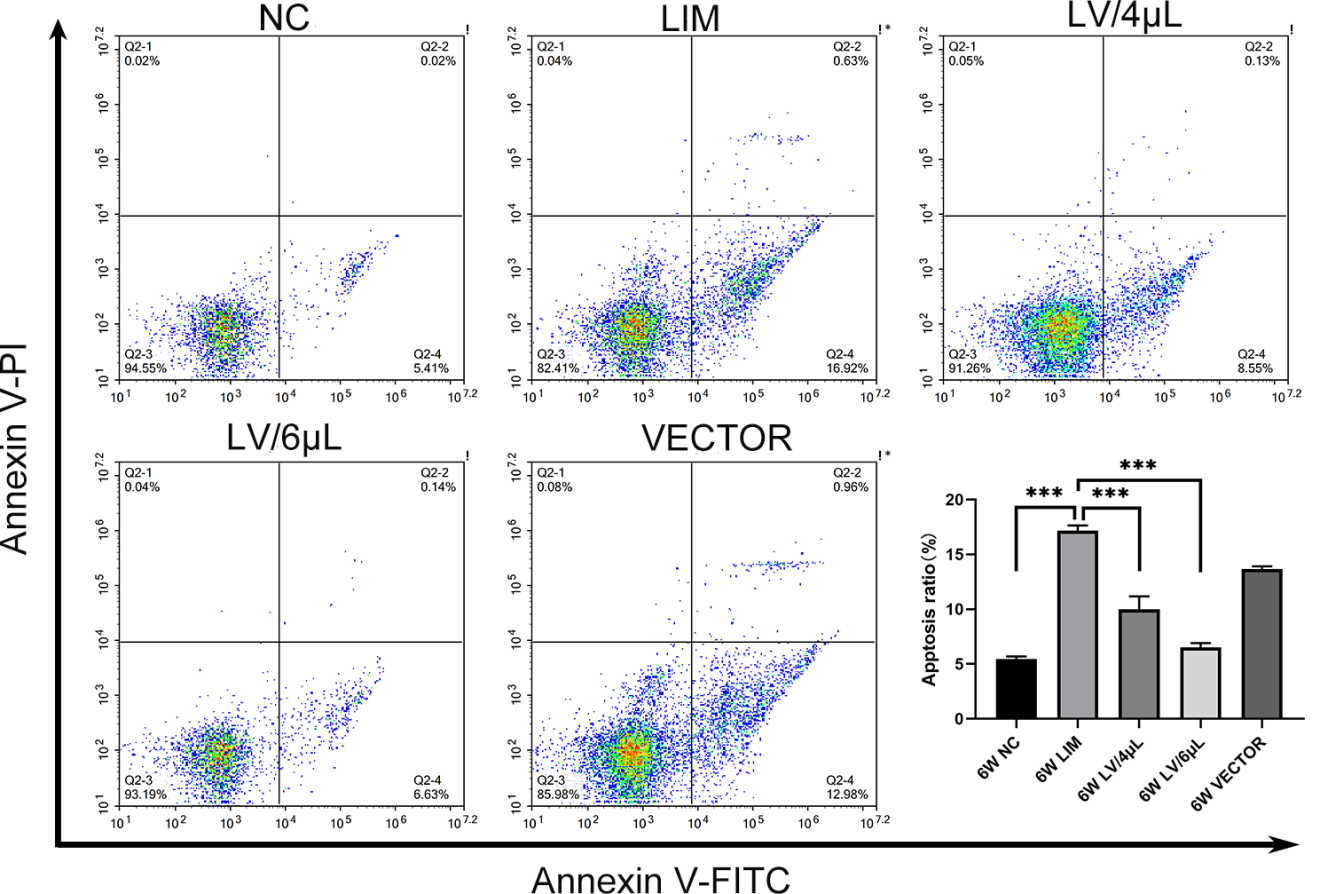
Levels of retinal cell apoptosis in each group were determined by flow cytometry. After 6 weeks of different treatments, retinal cell apoptosis was assessed using Annexin V/PI staining assays. *P < 0.05, **P < 0.01, and ***P < 0.001

We performed the retinal apoptosis assay using an Annexin V-FITC/PI apoptosis detection kit after 6-week miR-92b-3p treatments. The results showed that the type of apoptosis was mainly early apoptosis, and the apoptotic rate was 5.21% in the NC group, 16.65% in the LIM group, 8.68% in the LV/4µL group, 6.14% in the LV/6µL group, and 13.49% in the VECTOR group, respectively. The retinal cell apoptotic rate in the LIM group was significantly higher than that in the NC group, indicating that experimental myopia can aggravate retinal cell apoptosis. However, after treatment with miR-92b-3p-carring lentivirus, the retinal cell apoptotic rates in the LV/4µL group and the LV/6µL group were significantly lower than that of the LIM group, and the apoptotic rate in the LV/4µL group was higher than that of the LV/6µL group (Fig. 4). Our results indicated that experimental myopia can exacerbate retinal cell apoptosis, and miR-92b-3p effectively alleviates retinal cell apoptosis.

### Measurement of the mitochondrial membrane potential

**Fig. 5:**
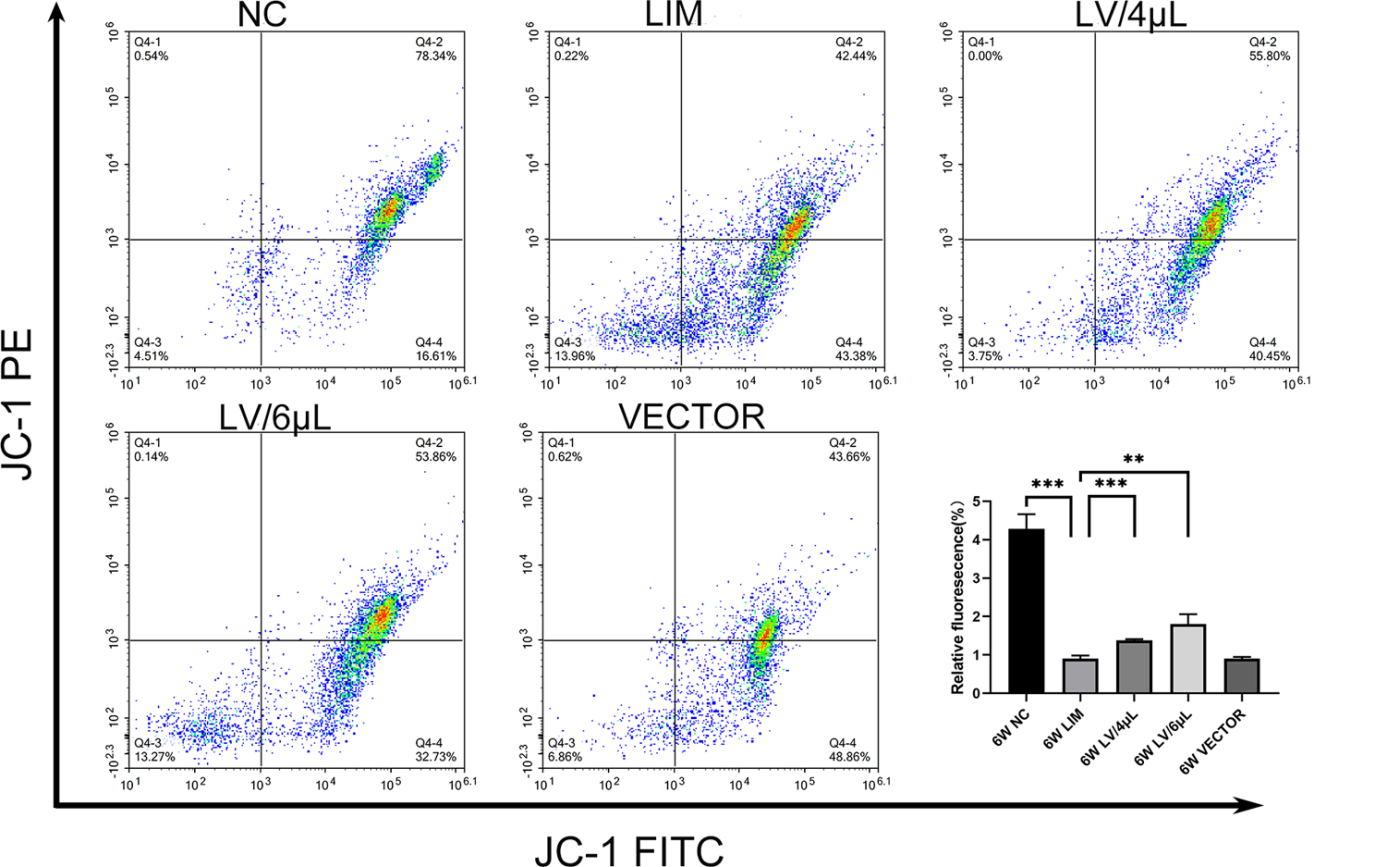
Changes in mitochondrial membrane potential in various groups. After 6 weeks of different treatments, JC-1 staining flow assay was used to assess the mitochondrial membrane potential of retinal cells. *P < 0.05, **P < 0.01, and ***P < 0.001

The mitochondrial membrane potential is high for normal cells. However, after 6-week myopic induction, we noted that the retinal cells were at the early stage of apoptosis, and their mitochondrial membrane potential was lower than that of the normal cells. By contrast, the mitochondrial membrane potential could be partially restored after 4µL and 6µL of miR-92b-3p treatments (Fig. 5), suggesting that myopia can disrupt the mitochondrial membrane potential, leading to mitochondrial dysfunction, whereas miR-92b-3p effectively improves the mitochondrial membrane potential, enhancing the mitochondrial functions.

### TUNEL assay

**Fig. 6:**
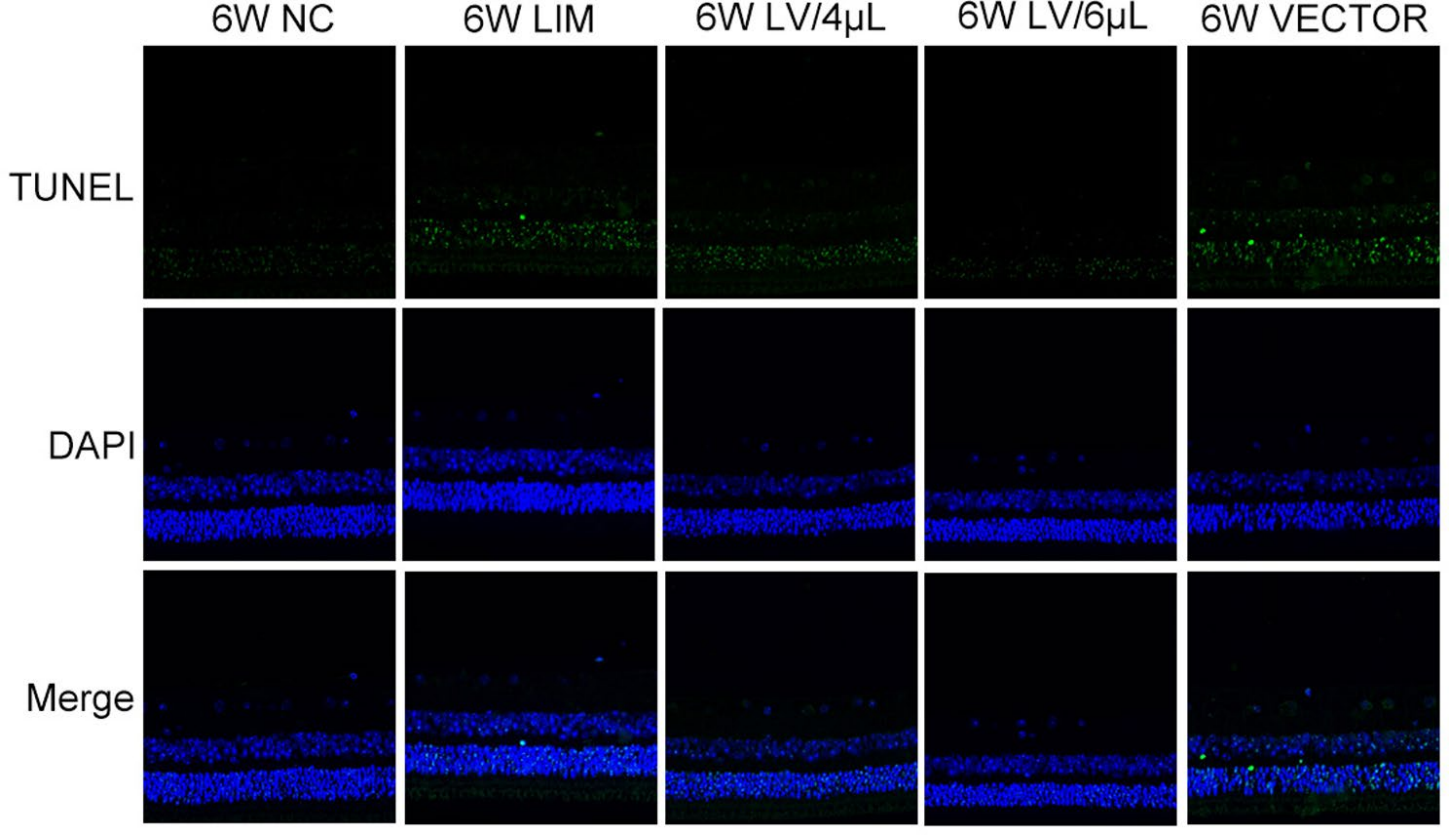
Apoptotic determination of retinal tissue of guinea pigs in each group. After 6-week different treatments, the eyeballs were isolated, and the TUNEL assay was performed to evaluate the retinal cell apoptosis. Magnification: 400×

We used TUNEL staining to explore the apoptosis of retinal cells after 6-week myopic induction. The results showed that the nuclei of DAPI-stained cells were blue, and the nuclei of DAB-stained apoptosis-positive cells were light green. After 6-week myopic induction, the green fluorescence signal intensity in the LIM group increased compared with that of the NC group. After treatment with either 4 µL or 6 µL of miR-92b-3p-carrying lentivirus, the green fluorescence signal intensity significantly decreased. Meanwhile, we also found that the green fluorescence signal intensity in the VECTOR group was similar to that of the LIM group (Fig. 6).

### qPCR analysis

**Fig. 7:**
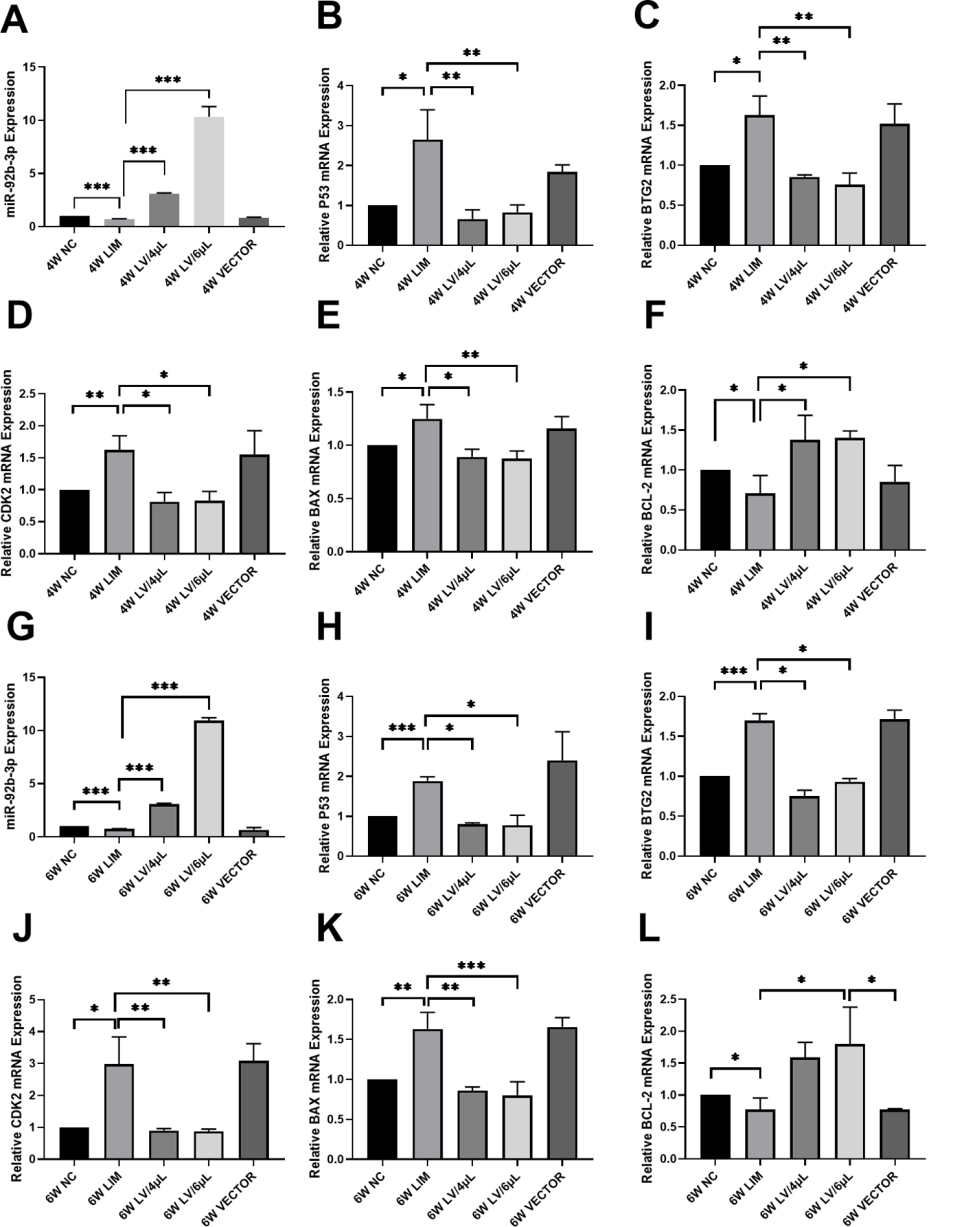
qPCR analysis of p53, BTG2, CDK2, BAX, BCL-2, and miR-92b-3p levels in retinal tissues after 4- and 6-week different treatments. Histogram analysis of miR-92b-3p (**A**, **G**), p53 (**B**, **H**), BTG2 (**C**, **I**), CDK2 (**D**, **J**), BAX (**E**, **K**), and BCL-2 (**F**, **L**) gene levels. *P < 0.05, **P < 0.01, and ***P < 0.001

To evaluate the effect of miR-92b-3p on apoptosis-related molecules, we measured the changes of p53, BTG2, CDK2, BAX, and BCL-2 gene levels in the retinal tissues after 4- and 6-week myopic induction. We found that p53, BTG2, CDK2, and BAX gene levels were significantly elevated in the LIM group, whereas the BCL-2 gene level decreased compared with the level of the NC group (Fig. 7, *P* < 0.05). By contrast, after treatment with either 4 µL or 6 µL of miR-92b-3p-carrying lentivirus, p53, BTG2, CDK2, and BAX gene levels significantly reduced compared with the levels of the LIM group, whereas the BCL-2 level (Fig. 7, all *P* < 0.05). In addition, we also noted that there was no significant difference between the LIM and VECTOR groups.

### Western blot analysis

**Fig. 8:**
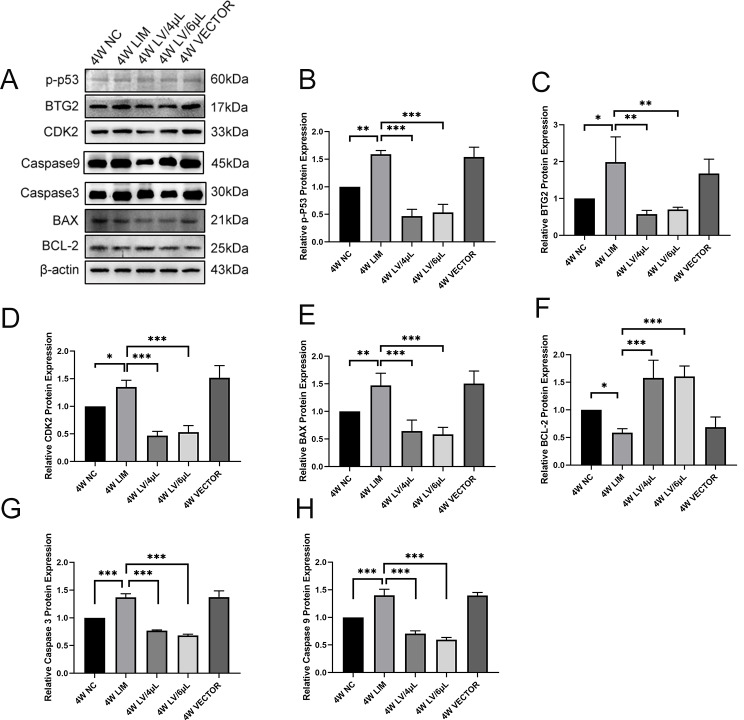
Western blot analysis of p-p53, BTG2, CDK2, Caspase 3, Caspase 9, BAX, and BCL-2 levels in the retinal tissues of guinea pigs in each group after 4-week different treatments. (**A**) Western blot, and histogram analysis of p-p53 (**B**), BTG2 (**C**), CDK2 (**D**), BAX (**E**), and BCL-2 (**F**), Caspase 3(**G**), Caspase 9(**H**). *P < 0.05, **P < 0.01, and ***P < 0.001

**Fig. 9:**
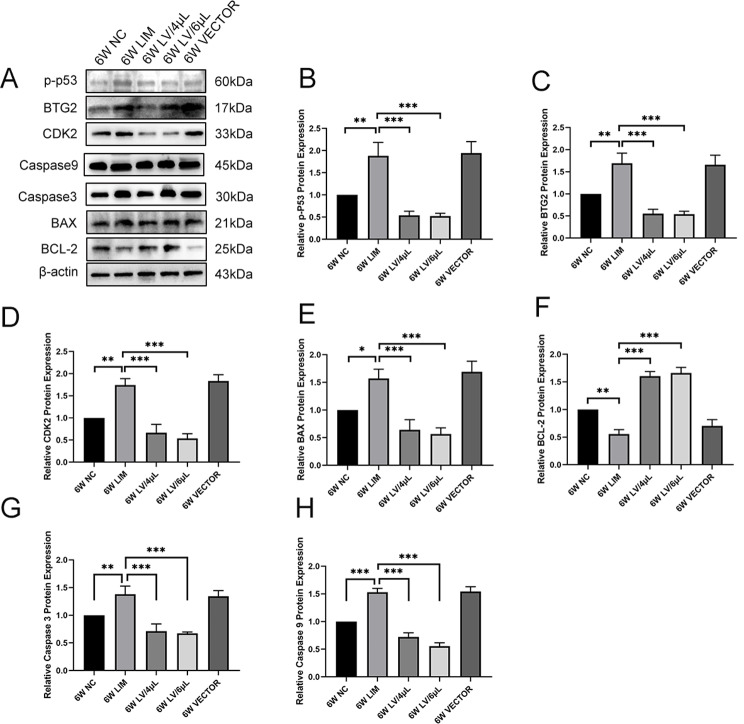
Western blot analysis of p-p53, BTG2, CDK2, Caspase 3, Caspase 9, BAX, and BCL-2 levels in the retinal tissues of guinea pigs in each group after 4-week different treatments. (**A**) Western blot, and histogram analysis of p-p53 (**B**), BTG2 (**C**), CDK2 (**D**), BAX (**E**), and BCL-2 (**F**), Caspase 3(*G*), Caspase 9(**H**). *P < 0.05, **P < 0.01, and ***P < 0.001

To investigate the change in the p53, BTG2, CDK2, BAX, and BCL-2 protein levels, we performed the Western blot analysis. The results showed that p53, BTG2, CDK2, and BAX protein levels in the LIM group were significantly increased compared with that of the NC group, whereas the BCL-2 level decreased (Fig. 8). After treatment with either 4 µL or 6 µL of miR-92b-3p-carrying lentivirus for 4 and 6 weeks, the p53, BTG2, CDK2, and BAX protein levels significantly decreased, whereas the BCL-2 level increased (Fig. 9).

### Immunofluorescence assay

**Fig. 10:**
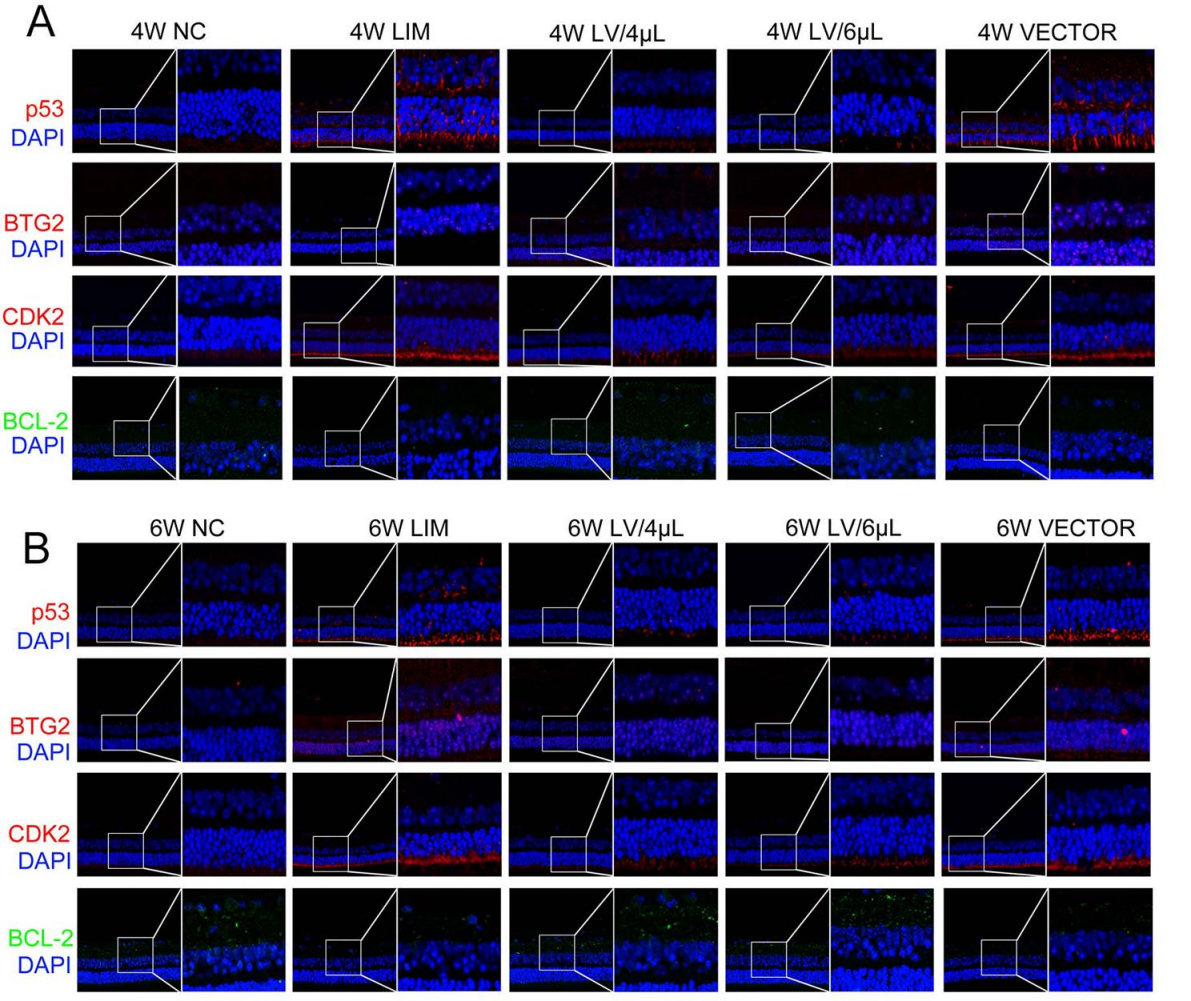
Comparison of p53, BTG2, BCL-2 and CDK2 expression in retinal tissues of NC, LIM, LV/4µL, LV/6µL, and VECTOR groups after myopic induction for 4 and 6 weeks. (**A**) Immunofluorescence staining after 4-week myopic induction, and (**B**) immunofluorescence staining after 6-week myopic induction. Magnification: 400×

As shown in Fig. 10, the results showed that the nuclei of DAPI-stained cells were blue under UV excitation, and the target protein-positive expression was fluorescein-labeled red. After 4-week myopic induction, the fluorescence intensity of p53, BTG2, BCL-2 and CDK2 in the retinal tissues of the guinea pigs was stronger than that of the NC group. However, after treatment with either 4 µL or 6 µL of miR-92b-3p-carrying lentivirus for 4 and 6 weeks, the expression of p53, BTG2 and CDK2 in the retinal tissues significantly decreased than those in the LIM group, and this result was identical to that of Western blot.

## Discussion

In the present study, OCT angiography showed a trend of thinning retinal thickness and a significant decrease in retinal electroretinogram dMax-a and -b wave amplitudes in the LIM guinea pigs, indicating the disrupted retinal electrophysiological function. Meanwhile, we also found that BTG2 could regulate the expression of CDK2, BAX, and BCL-2 downstream molecules related to BTG2 signaling; meanwhile, it can also regulate the expression of upstream molecule p53 in a negative feedback manner, resulting in the onset of DNA damage pathway in retinas of myopic guinea pigs and initiating apoptosis, demonstrating a disturbed retinal microenvironment. Supplement of the miR-92b-3p can protect the retinal tissues against DNA damage and apoptosis, and maintain the balance of the retinal microenvironment, thereby retarding the progression of myopia.

DNA is unstable and is prone to strand breaks during replication when disturbed by the external environment and intracellular metabolites [20]. It has been shown that BTG2 is associated with DNA damage, and its expression significantly increased in response to DNA damage [21, 22]. Importantly, the increase in BTG2 expression mediated by DNA damage is due to p53 induction [11]. p53 plays a key role as a tumor suppressor, activated in response to DNA damage, hypoxia, or conditions that prevent cell cycle progression and thus maintain genomic integrity [23]. It has been reported that the protection of cardiomyocytes from oxidative stress, DNA damage, and mitochondrial dysfunction induced by H_2_O_2_ exposure is involved in the regulation of the p53-BTG2 signaling pathway [24]. This finding also suggests a close relationship between p53-BTG2 and DNA damage. DNA instability and defective DNA repair are also associated with the onset and progression of retinal neuronal degeneration [25]. In our study, experimental myopia can induce an increase in BTG2 and p53 gene expression, indicating that sustained myopia induction leads to the exaggeration of DNA damage. In addition, BTG2-mediated apoptosis is accompanied by modification of p53 protein phosphorylation, usually due to post-translational modification of p53 by BTG2 through regulation of BAX expression [26]. Considering that miR-92b-3p can negatively regulate the BTG2 expression, thus, the supplement of miR-92b-3p can decrease the BTG2 level, thereby causing the attenuated p-p53 expression level (Fig. 7) and protecting the retinal tissue against internal DNA damage.

Cell cycle protein-dependent kinase 2 (CDK2) plays a crucial role in cell cycle regulation and is involved in a range of biological processes, such as DNA damage, intracellular translocation, protein degradation, signal transduction, DNA and RNA metabolism, and translation [27], and CDK2-mediated DNA damage is mainly due to the accumulation of p53 and CDK2-induced cell cycle arrest [28]. However, CDK2 activation largely disrupts DNA replication and leads to DNA replication stress, which may refer to the slowing or stalling of replication fork processes during DNA synthesis in response to different injuries, ultimately inducing the generation of apoptosis [29]. In the present study, continuous myopia induction will lead to increased CDK2 expression, and this continuous external stimulation causes sustained DNA damage to the retina. With the accumulation of DNA damage, DNA damage will be too severe to be repaired. Consequently, p53 will promote apoptosis by activating the mitochondrial apoptotic pathway to damage the retinal physiological function [30], thereby leading to a decrease in retinal electroretinogram dMax-a and -b wave amplitudes.

MicroRNAs (miRNAs) can interact with target mRNAs at specific sites to induce cleavage of the target mRNAs or inhibit translation. miR-92b-3p is involved in several biological processes such as proliferation, migration, apoptosis, and angiogenesis [31]. It has been demonstrated that miR-92b-3p regulates cell cycle and apoptosis by targeting CDKN1C and affects the sensitivity of cells to chemotherapeutic agents [32]. Based on bioinformatics annotation, miR-92b-3p can negatively regulate the BTG2 expression. To further elucidate the mechanism of BTG2 regulated by miR-92b-3p, we verified the binding sequence of BTG2 to miR-92b-3p by several databases (mirDIP, miRDB, and Targetscan) and combined with dual luciferase reporter assay, and performed GO enrichment for BTG2 upstream and downstream genes. We found that the level of miR-92b-3p in the retina was negatively correlated with BTG2 in the LIM guinea pigs, and the GO enrichment results also showed a correlation with the biological processes of DNA damage and apoptosis. We further verified this prediction and found that miR-92b-3p can negatively regulate the BTG2 expression in the retina in LIM guinea pigs, the supplement of miR-92b-3p can effectively decrease the BTG2 level, ameliorate DNA damage and apoptosis in the retina of LIM guinea pigs. Our previous study has revealed that the degree of fibrosis in the choroidal tissue of myopic guinea pigs significantly reduced after the miR-138-5p supplement, and the ocular axial length decreased. These findings indicate that miRNAs may be a useful target in delaying myopia progression [33]. 

**Fig. 11:**
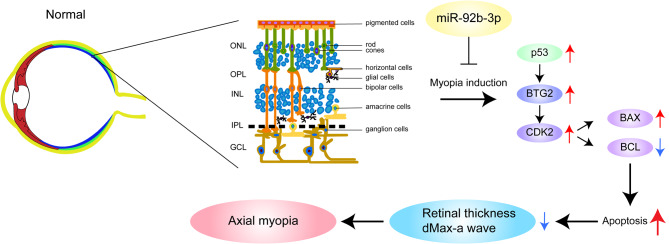
Intravitreal injection of miR-92b-3p attenuated the elongation of axial length and retinal thickness, and inhibited the expression of CDK2, BAX, and p53 by targeting BTG2, thereby ameliorating DNA damage and apoptosis in LIM guinea pigs and protecting ocular tissues

Chen and colleagues reported that blue light can induce DNA double-strand breaks in retinal neurons, impairing the viability of retinal nerve cells and inducing apoptosis [34]. Similarly, the transcriptomic analysis also revealed that p53 can mediate apoptosis of retinal pigment epithelial cells and is correlated with DNA damage, inducing downstream BAX and BCL-2 expression [35]. In our study, we found that the type of apoptosis was mainly early apoptosis, and the mitochondrial membrane potential was reduced in the LIM animals, indicating that LIM could disrupt the physiological function of the retinal cells. miR-92b-3p effectively alleviates retinal cell apoptosis and restores the mitochondrial membrane potential. We also found that decreased miR-92b-3p caused elevated BTG2, CDK2, and p53. p53 mainly accumulated in the pigment epithelium of the retina, indicating that p53 can also participate in DNA damage and repair processes. In addition, the TUNEL assay confirms that apoptosis could also occur in the retinal tissue of the myopic eye, and a decrease in dMax-a and -b wave amplitude indicates impaired retinal function. In contrast, a supplement of miR-92b-3p could inhibit the expression of btg2, cdk2, and p53, attenuate retinal tissue-related cell apoptosis, enhance the retinal thickness and improve retinal physiological function, thereby suppressing the development of experimental myopia (Fig. 11).

However, there are still limitations in the current study. Firstly, we did not knock down the BTG2 gene to observe the effect of the lack of BTG2 on DNA damage- and apoptosis-related genes as well as the development of myopia. In addition, we need to investigate the detailed mechanism of whether DNA damage related to the myopic retina affects the physiological functions of the choroid and sclera. Nevertheless, our results still provide a new idea for the subsequent myopia treatment.

## Conclusion

In conclusion, our study confirmed that negative lens-induced myopia can decrease the miR-92b-3p expression, enhance the BTG2 level, and induce DNA damage of retinal tissues of guinea pigs, causing apoptosis of retinal tissue, resulting in thinning retinal thickness and disrupted electrophysiological function, which is involved in the development of axial myopia. Supplement of miR-92b-3p effectively attenuates the retinal apoptosis caused by DNA damage, suppresses retinal apoptosis, enhances the retinal thickness, and restores the retinal electrophysiological function, thus inhibiting myopia progression. Our results indicate that BTG2-mediated apoptosis and retinal damage may be a potential target in the treatment of myopia. miR-92b-3p may play an important role in the treatment of myopia, providing a new idea for the treatment of myopia.

## Data Availability

The datasets used and/or analyzed during the current study are available from the corresponding author on reasonable request.

## Abbreviations

NC: Normal control
LIM: Lens-introduced myopia
FERG: Flash electroretinogram(
3’UTR: 3’ Untranslated region
BTG2: BTG Anti-Proliferation Factor 2
CDK2: Cyclin Dependent Kinase 2
OCT: Optical Coherence Tomography
LV/4µL: LIM + miR-92b-3p-carrying lentivirus (4µL) treatment group
LV/6µL: LIM + miR-92b-3p-carrying lentivirus (6µL) treatment
DAPI: 4′6-diamidino-2-phenylindole
TUNEL: TdT-mediated dUTP-biotin nick labeling
GCL: Ganglion cell layer
IPL: Inner plexiform layer
INL: Inner nuclear layer
OPL: Outer plexiform layer
ONL: Outer nuclear layer

## References

[1] Morgan IG (2018). The epidemics of myopia: Aetiology and prevention. Prog Retin Eye Res.

[2] Li SM (2022). Annual incidences and progressions of Myopia and High Myopia in Chinese Schoolchildren based on a 5-Year Cohort Study. Invest Ophthalmol Vis Sci.

[3] Harb EN, Wildsoet CF (2019). Origins of refractive errors: environmental and genetic factors. Annu Rev Vis Sci.

[4] Hoon M, Okawa H, Della Santina L, Wong RO (2014). Functional architecture of the retina: development and disease. Prog Retin Eye Res.

[5] Yang GY, et al. Associations between screen exposure in early life and myopia amongst Chinese preschoolers. Int J Environ Res Public Health. 2020;17. 10.3390/ijerph17031056. 10.3390/ijerph17031056 PMC7037286 32046062

[6] Blasiak J, Szaflik JP (2011). DNA damage and repair in age-related macular degeneration. Front Biosci (Landmark Ed).

[7] Barzilai A, Biton S, Shiloh Y (2008). The role of the DNA damage response in neuronal development, organization and maintenance. DNA Repair (Amst).

[8] Sasaki M (2012). Biological role of lutein in the light-induced retinal degeneration. J Nutr Biochem.

[9] Mao B, Zhang Z, Wang G (2015). BTG2: a rising star of tumor suppressors (review). Int J Oncol.

[10] Matsuda S, Rouault J, Magaud J, Berthet C (2001). In search of a function for the TIS21/PC3/BTG1/TOB family. FEBS Lett.

[11] Rouault JP (1996). Identification of BTG2, an antiproliferative p53-dependent component of the DNA damage cellular response pathway. Nat Genet.

[12] Bartel DP (2004). MicroRNAs: genomics, biogenesis, mechanism, and function. Cell.

[13] Hu JL (2019). CAFs secreted exosomes promote metastasis and chemotherapy resistance by enhancing cell stemness and epithelial-mesenchymal transition in colorectal cancer. Mol Cancer.

[14] Jalava SE (2012). Androgen-regulated miR-32 targets BTG2 and is overexpressed in castration-resistant prostate cancer. Oncogene.

[15] Zhang BG (2012). microRNA-21 promotes tumor proliferation and invasion in gastric cancer by targeting PTEN. Oncol Rep.

[16] Zhou Y (2019). Metascape provides a biologist-oriented resource for the analysis of systems-level datasets. Nat Commun.

[17] Zhou X (2006). Normal development of refractive state and ocular dimensions in guinea pigs. Vis Res.

[18] Ferguson LR, Dominguez JM, Balaiya S, Grover S, Chalam KV (2013). Retinal thickness normative data in wild-type mice using customized miniature SD-OCT. PLoS ONE.

[19] Livak K J, Schmittgen D T (2001). Analysis of relative gene expression data using real-time quantitative PCR and the 2(-Delta Delta C(T)) method. Methods.

[20] Murray JM, Carr AM (2018). Integrating DNA damage repair with the cell cycle. Curr Opin Cell Biol.

[21] Kis E (2006). Microarray analysis of radiation response genes in primary human fibroblasts. Int J Radiat Oncol Biol Phys.

[22] Chu TY, Yang JT, Huang TH, Liu HW (2014). Crosstalk with cancer-associated fibroblasts increases the growth and radiation survival of cervical cancer cells. Radiat Res.

[23] Levine AJ (1997). p53, the cellular gatekeeper for growth and division. Cell.

[24] Wang K, Zhu QZ, Ma XT, Cheng C (2021). SUV39H2/KMT1B inhibits the cardiomyocyte senescence phenotype by down-regulating BTG2/PC3. Aging.

[25] Zou M (2022). Inhibition of cGAS-STING by JQ1 alleviates oxidative stress-induced retina inflammation and degeneration. Cell Death Differ.

[26] Choi OR, Ryu MS, Lim IK (2016). Shifting p53-induced senescence to cell death by TIS21(/BTG2/Pc3) gene through posttranslational modification of p53 protein. Cell Signal.

[27] Tadesse S (2020). Targeting CDK2 in cancer: challenges and opportunities for therapy. Drug Discov Today.

[28] Shieh SY, Ahn J, Tamai K, Taya Y, Prives C (2000). The human homologs of checkpoint kinases Chk1 and Cds1 (Chk2) phosphorylate p53 at multiple DNA damage-inducible sites. Genes Dev.

[29] Fagundes R, Teixeira LK, Cyclin (2021). E/CDK2: DNA replication, replication stress and genomic instability. Front Cell Dev Biol.

[30] Roos WP, Kaina B (2013). DNA damage-induced cell death: from specific DNA lesions to the DNA damage response and apoptosis. Cancer Lett.

[31] Liang G (2021). MiR-92b-3p inhibits proliferation of HER2-Positive breast Cancer Cell by Targeting circCDYL. Front Cell Dev Biol.

[32] Zhao F, et al. miR-92b-3p regulates cell cycle and apoptosis by targeting CDKN1C, thereby affecting the sensitivity of Colorectal Cancer cells to chemotherapeutic drugs. Cancers (Basel). 2021;13. 10.3390/cancers13133323. 10.3390/cancers13133323 PMC8268555 34283053

[33] Li T (2023). Inhibitory effect of mir-138-5p on choroidal fibrosis in lens-induced myopia guinea pigs via suppressing the HIF-1alpha signaling pathway. Biochem Pharmacol.

[34] Chen P, et al. Retinal neuron is more sensitive to Blue Light-Induced damage than Glia Cell due to DNA double-strand breaks. Cells. 2019;8. 10.3390/cells8010068. 10.3390/cells8010068 PMC6356720 30669263

[35] Lin YC, Shen ZR, Song XH, Liu X, Yao K (2018). Comparative t ranscriptomic analysis reveals adriamycin-induced apoptosis via p53 signaling pathway in retinal pigment epithelial cells. J Zhejiang Univ Sci B.

